# Antibody mediated activation of natural killer cells in malaria exposed pregnant women

**DOI:** 10.1038/s41598-021-83093-4

**Published:** 2021-02-18

**Authors:** Timon Damelang, Elizabeth H. Aitken, Wina Hasang, Ester Lopez, Martin Killian, Holger W. Unger, Ali Salanti, Alexis Shub, Elizabeth McCarthy, Katherine Kedzierska, Martha Lappas, Stephen J. Kent, Stephen J. Rogerson, Amy W. Chung

**Affiliations:** 1 Department of Microbiology and Immunology, Peter Doherty Institute for Infection and Immunity, University of Melbourne, Melbourne, VIC Australia; 2 Department of Medicine, Peter Doherty Institute for Infection and Immunity, University of Melbourne, Melbourne, VIC Australia; 3 Department of Internal Medicine, Centre Hospitalier Universitaire de Saint-Etienne, Saint-Etienne, France; 4 Groupe sur l’Immunité des Muqueuses et Agents Pathogènes, Université Jean Monnet Saint-Etienne, Saint-Etienne, France; 5 Liverpool School of Tropical Medicine, Liverpool, UK; 6 Department of Obstetrics and Gynaecology, Royal Darwin Hospital, Darwin, NT Australia; 7 Menzies School of Health Research, Charles Darwin University, Darwin, NT Australia; 8 Department for Immunology and Microbiology, Centre for Medical Parasitology, University of Copenhagen, Copenhagen, Denmark; 9 Department of Infectious Disease, Copenhagen University Hospital, Copenhagen, Denmark; 10 Department of Obstetrics and Gynaecology, University of Melbourne, Melbourne, VIC Australia; 11 Infectious Diseases Department, Alfred Health, Melbourne Sexual Health Centre, Monash University, Melbourne, VIC Australia

**Keywords:** Immunology, Diseases

## Abstract

Immune effector responses against *Plasmodium falciparum* include antibody-mediated activation of innate immune cells, which can induce Fc effector functions, including antibody-dependent cellular cytotoxicity, and the secretion of cytokines and chemokines. These effector functions are regulated by the composition of immunoglobulin G (IgG) Fc *N*-linked glycans. However, a role for antibody-mediated natural killer (NK) cells activation or Fc *N*-linked glycans in pregnant women with malaria has not yet been established. Herein, we studied the capacity of IgG antibodies from pregnant women, with placental malaria or non-placental malaria, to induce NK cell activation in response to placental malaria-associated antigens DBL2 and DBL3. Antibody-mediated NK cell activation was observed in pregnant women with malaria, but no differences were associated with susceptibility to placental malaria. Elevated anti-inflammatory glycosylation patterns of IgG antibodies were observed in pregnant women with or without malaria infection, which were not seen in healthy non-pregnant controls. This suggests that pregnancy-associated anti-inflammatory Fc *N*-linked glycans may dampen the antibody-mediated activation of NK cells in pregnant women with malaria infection. Overall, although anti-inflammatory glycans and antibody-dependent NK cell activation were detected in pregnant women with malaria, a definitive role for these antibody features in protecting against placental malaria remains to be proven.

## Introduction

**Figure 1:**
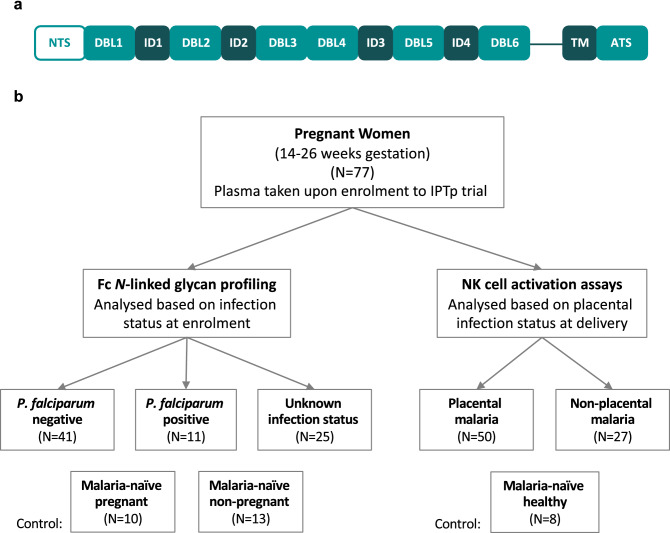
Schematic representation of VAR2CSA and overview of cohort groups. (**a**) The extracellular region of VAR2CSA contains a N-terminal sequence (NTS) followed by Duffy binding-like (DBL) domains and interdomain (ID) regions. It is anchored in the membrane by a transmembrane (TM) domain connected to an acidic terminal segment (ATS). (**b**) Plasma samples were obtained from pregnant women in Papua New Guinea between November 2009 and August 2012 upon enrolment into an Intermittent Preventive Treatment in Pregnancy (IPTp) randomized controlled trial at 14–26 gestation weeks. Samples were grouped based on infection status at enrolment for Fc *N*-linked glycan profiling and grouped based on infection status at delivery for functional NK cell activation assays.

*Plasmodium falciparum*, the main causative agent of malaria, poses a serious threat to the health of pregnant women and to their unborn babies. Malaria in pregnant women can not only cause maternal death and life-threatening symptoms, such as anemia, pulmonary edema, hypoglycemia, puerperal sepsis, but also miscarriages, stillbirths, prematurity and fetal growth restriction^1^. Globally, malaria contributes to more than 20% of all maternal deaths in malaria endemic areas^1^. Pregnant women are more susceptible to malaria than their non-pregnant counterparts^2^, not only due to immunological changes during pregnancy, but also to the unique characteristics of *P. falciparum* parasites that can accumulate and sequester in the maternal blood spaces of the placenta^3,4^. In placental malaria, a single member of the *P. falciparum* erythrocyte membrane protein 1 (*Pf*EMP1) family^5,6^, called VAR2CSA, is expressed on *P. falciparum*-infected erythrocytes (IEs), and mediates adhesion to the glycosaminoglycan chondroitin sulphate A (CSA), on the syncytiotrophoblast cell surface lining of the maternal blood spaces^7–10^. This adhesion avoids splenic clearance of IEs from the blood circulation, which leads to inflammation and localized endothelial dysfunction of the placenta^11^. The variant surface antigen VAR2CSA is a large ~ 350 kDa transmembrane protein consisting of a cytoplasmic acidic terminal segment, six extracellular Duffy binding-like (DBL) domains, four inter-domain (ID) regions, and a N-terminal segment^12,13^ (Fig. 1a).

Whilst antibody (Ab) responses to several VAR2CSA domains are positively associated with the presence of placental and peripheral infections, there is little evidence that Ab levels to recombinant proteins protect from placental malaria^14^. However, studies have shown that immunoglobulin G (IgG) Abs recognize VAR2CSA in a sex-specific and parity-dependent manner^10^. High anti-VAR2CSA IgG levels can be found in multigravid pregnant women in *P. falciparum*-endemic regions^15^, and women with high plasma levels of anti-VAR2CSA IgG have a decreased risk of delivering low-birthweight babies^10^. The predominant IgG subclasses produced in response to *P. falciparum* in pregnancy are IgG1 and IgG3^15–17^. Both IgG subclasses have been linked to protective immunity against *P. falciparum* infections^18,19^, possibly due to opsonization of IEs and the Ab-mediated activation of Fc gamma receptor (FcγR) expressing innate immune cells including phagocytes and natural killer (NK) cells^20,21^.

NK cells can mediate Ab-dependent cellular cytotoxicity (ADCC) upon recognition of target cells via FcγRIIIa^22^, which is hypothesized to play a possible role in direct cytotoxic killing of IEs, and therefore is suggested to be beneficial against *P. falciparum* infections^23^. Ab-mediated activation of NK cells can also induce the secretion of a range of cytokines, including interferon gamma (IFNγ) and tumor necrosis factor alpha (TNFα)^24–26^. These cytokines may be beneficial during the early phase of *Plasmodium* infection by reducing parasitemia^22,23^*.* However, overproduction of pro-inflammatory cytokines can also result in immunopathology and adverse clinical outcomes, especially in pregnancy^27–29^.

Antigen-specific Ab engagement with FcγRIIIa on NK cells was recently identified as a key vaccine-induced functional immune responses linked to protection by RTS,S/AS01, the only licensed *P. falciparum* vaccine^30^. In addition, in vitro assays demonstrated the ability of NK cells to kill IEs via ADCC, and IgG Abs to *Pf*EMP1 were sufficient to promote NK-dependent growth inhibition of *P. falciparum* in IEs^31^. This study also showed that naturally acquired IgG of multigravid women specific for VAR2CSA promotes NK-dependent lysis of IEs^31^. The ability of IgG Abs against the DBL2 and its flanking ID regions of VAR2CSA to induce ADCC is still unexplored^32^, but is of special interest, since the two leading placental malaria vaccine candidates PRIMVAC (Institut National de la Santé et de la Recherche Médicale, France) and PAMVAC (University Hospital Tuebingen, Germany) both include DBL2 domains^33,34^.

Fc effector functions such as ADCC are regulated through multiple structural and genetic components of the Ab, FcγR, and effector cell^35^, including post-translational modifications of glycans on the Fc domain of Abs, specifically at asparagine 297 on IgG^36^. Multiple factors can influence glycosylation patterns of IgG Abs including age, sex^37^, epigenetics^38^, disease state^39,40^, infection^41–43^, or vaccination^44^. Glycosylation patterns of IgG Abs can also undergo temporary changes during pregnancy, when galactosylation and sialylation of IgG Abs increase^45,46^. This has been associated with a less inflammatory profile^47^, which may contribute to acceptance of the placenta by the maternal immune system during pregnancy^48,49^. Changes in the composition of the asparagine 297 glycan can also influence the binding affinity of IgG Abs to FcγRs, and thereby change the magnitude of effector functions initiated, including ADCC and Ab-dependent cellular phagocytosis^50^. Human NK cells primarily express one Fc gamma receptor (FcγRIIIa), and responses through FcγRIIIa are highly regulated by IgG *N*-linked glycosylation, more so than any other human FcγR^51–53^. Some studies suggest that the presence/absence of key glycoforms can modulate FcγR affinity and ADCC activity by up to 20-fold^36,51,54,55^.

Here, we investigate the ability of IgG Abs of pregnant women from a malaria-endemic area specific to DBL2 and DBL3 (both VAR2CSA domains) to activate human primary NK cells from malaria-naïve donors to secrete IFNγ and TNFα cytokines, and upregulate CD107a expression, which is a surrogate for granzyme B degranulation and ADCC activity^56^. In addition, we evaluated pregnancy-associated glycosylation patterns of IgG Abs and their effect on NK-mediated effector functions in the context of *P. falciparum* infection during pregnancy.

## Results

### Primary human NK cells are activated by DBL2 or DBL3-specific IgG Abs from pregnant women with malaria

NK cells are major innate immune mediators of cytotoxicity. To evaluate the capacity of DBL2 and DBL3-specific IgG Abs to induce NK-mediated effector functions, we used purified IgG from two groups of pregnant women at mid pregnancy with peripheral *P. falciparum* parasitemia at delivery, and who were either positive (N = 50) or negative for *P. falciparum* IEs in the placenta (N = 27) (Fig. 1b).

**Figure 2:**
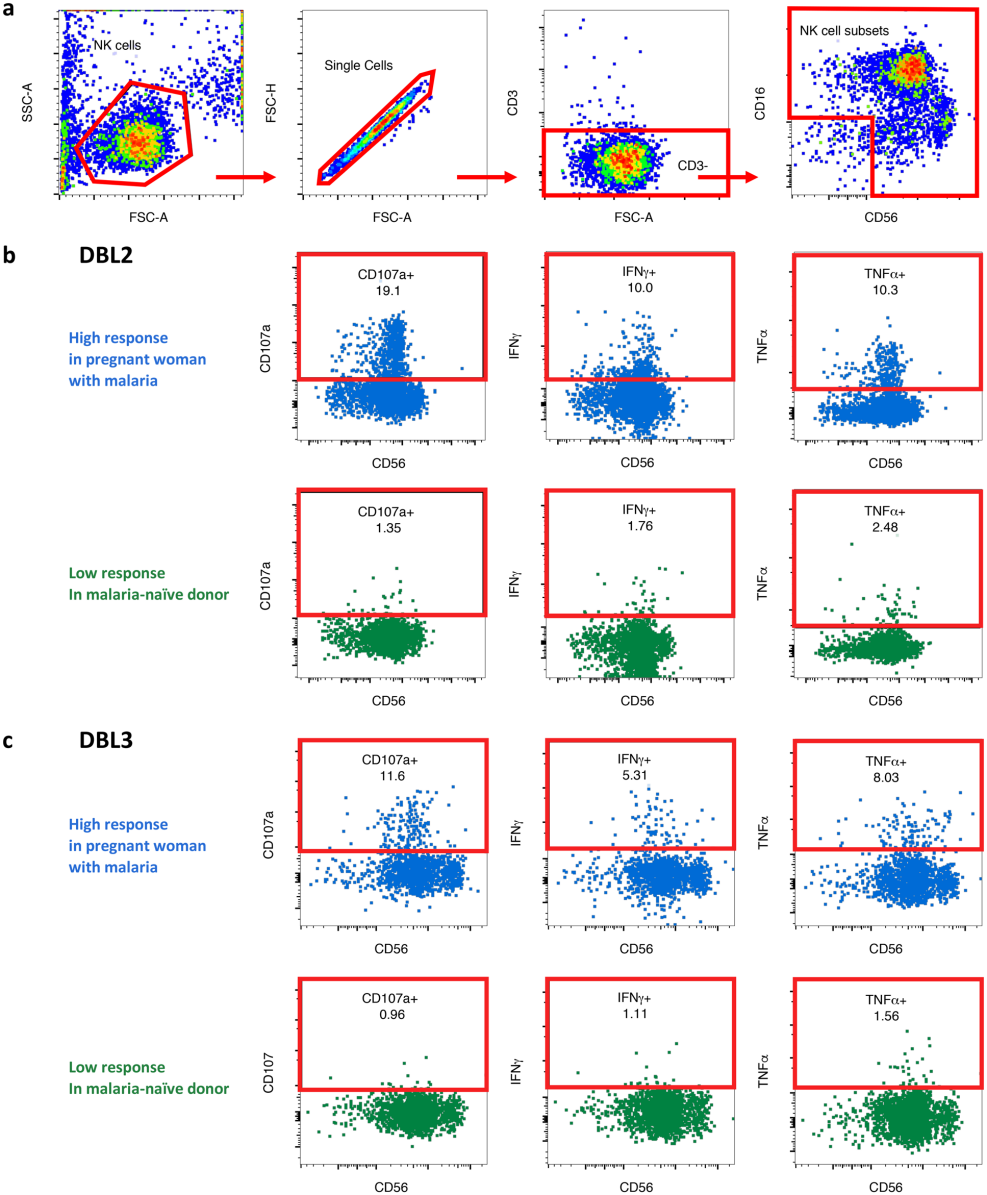
Gating strategy to identify NK cell activation markers. Purified NK cells were incubated with IgG test samples in presence of DBL2 or DBL3 for 5 h and then analyzed via flow cytometry. Representative flow cytometry plots of one sample to visualize gating strategy. (**a**) NK cells were identified by sequentially gating on lymphocytes, single cells, CD3^-^ cells, and NK cell subsets. NK cells subsets were gated as one and assessed for surface CD107a expression and intracellular IFNγ and TNFα production in presence of DBL2 (**b**) and DBL3 (**c**) (High response = blue; malaria-naïve response = green).

We modified previously described Ab-dependent NK cell activation assays that have been utilized to assess responses to influenza, human immunodeficiency virus (HIV) and *Mycobacterium tuberculosis* proteins^24–26,57^ for the use with VAR2CSA domain antigens (Fig. 1a). DBL2 was chosen because of its relevance in the development of placental malaria vaccines^33,34^. DBL3 is another domain of the VAR2CSA protein, which can be recognized by IgG Abs generated by pregnant women with malaria^58^. We characterized the ability of Abs against these domains to activate primary human NK cells, isolated from the blood of three malaria-naïve healthy donors. NK cells were identified via flow cytometry (Fig. 2a) and the levels of Ab-mediated NK cell activation in response to DBL2 and DBL3 were measured as indicated by intracellular cytokine production of IFNγ and TNFα, and the upregulation of cell surface degranulation marker CD107a (Fig. 2b-c). In order to optimize the Ab-dependent NK cell activation assay for malaria antigens, DBL2 (50–300 ng/well), DBL3 (50–300 ng/well) and IgG Ab (0.0625–0.25 mg/ml) concentrations were first titrated using four individual control Ab samples from pregnant women with malaria and a malaria-naïve individual (Fig. S1a–d).

**Figure 3:**
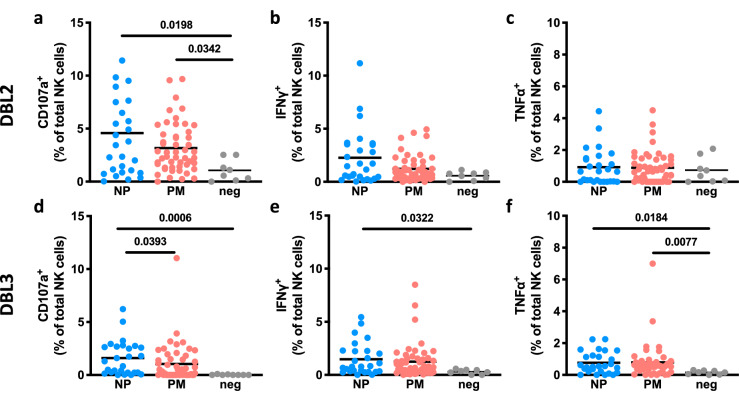
Human NK cells lack activation in presence of DBL2- or DBL3-specific Abs from pregnant women with malaria. NK cells were assessed for surface CD107a expression and intracellular IFNγ and TNFα production in the presence of VAR2CSA subdomains DBL2 (**a**–**c**) or DBL3 (**d**–**f**). Percentage of activation markers expressed by NK cells (mean of three separate donors) are shown. NK cells were stimulated with purified IgG Abs from pregnant women mid pregnancy with placental malaria (PM; N = 50; red) or from pregnant women with non-placental malaria (NP; N = 27; blue) at delivery in the presence of VAR2CSA subdomains DBL2 or DBL3. IgG Abs from malaria-naïve donors were used as negative control (N = 8; grey). Activation marker expression of NK cells incubated without Abs and median of SIV gp120-specific responses were subtracted as background. Statistical comparison between groups was performed using a Kruskal–Wallis test corrected for multiple comparisons using Dunn’s multiple comparison method (p-values are shown on graphs).

We evaluated purified IgG from pregnant women at mid pregnancy with peripheral *P. falciparum* parasitemia at delivery, and who were either positive or negative for *P. falciparum* IEs in the placenta. Their purified IgG was assessed in the presence of DBL2 or DBL3 for induction of Ab-mediated NK cell activation (Fig. 3). For both antigens, we observed upregulation of NK cell degranulation (CD107a; Fig. 3a,d, DBL2: p-value = 0.0198, DBL3: p-value = 0.0006) mid pregnancy in pregnant women who have non-placental malaria at delivery compared to non-pregnant malaria-naïve healthy individuals. In addition, DBL3-specific IgG mid pregnancy from pregnant women with non-placental malaria at delivery induced significantly higher IFNγ and TNFα production (Fig. 3e,f, IFNγ: p-value = 0.0322, TNFα: p-value = 0.0184) compared to IgG from non-pregnant malaria-naïve healthy individuals. Relative to IgG from non-pregnant malaria-naïve healthy individuals, IgG from pregnant women with placental malaria were associated with significantly higher NK cell degranulation (CD107a upregulation; Fig. 3a, p-value = 0.0342) in response to DBL2 and TNFα production (Fig. 3f, p-value = 0.0077) in response to DBL3 antigen. Differences in NK cell degranulation or cytokine production between pregnant women with non-placental malaria and women with placental malaria were only observed in CD107a expression (Fig. 3d, p-value = 0.0393) in response to DBL3.

Ab-dependent NK cell activation assays were validated with the use of two control antigens. In the presence of IgG Abs from malaria-naïve healthy individuals, influenza H3 (positive control) induced NK cell activation^26^, whereas the negative control SIV gp120 did not (Fig. S2a–c). Using human primary NK cells from malaria-naïve healthy donors, H3-specific Abs induced significant CD107a upregulation and cytokine expression (median expression [interquartile range (IQR)]: CD107a: 11.1% [10.2–13.1%], IFNγ: 7.0% [5.1–10.8%], TNFα: 6.2% [3.9–6.7%]), whereas SIV gp120 did not (median expression [IQR]: CD107a: 1.3% [0.7–1.6%], IFNγ: 0.3% [0.1–0.7%], TNFα: 0.0% [0.0–0.3%]). Overall, weaker NK cell degranulation or expression of intracellular cytokines IFNγ and TNFα were observed in the presence of DBL2 and DBL3-specific Abs compared to H3-specific Abs (Figs. 3 and S2). The mean of activation markers expressed by NK cells in the presence of IgG-coated DBL2 or DBL3 never reached the expression observed in the presence of influenza H3-specific IgG.

In addition, DBL2- and DBL3-binding capacities of IgG1-4 subclasses from pregnant women with placental (N = 50) and non-placental malaria (N = 27) were investigated via multiplex assays and correlated to the expression of CD107a, IFNγ and TNFα by Ab-activated NK cells (Figs. S3 and S4). The majority of non-placental malaria Ab-mediated NK cell activation was driven by IgG1; however, these correlations, if present, were weak to moderate (max Spearman ρ = 0.553, p-value = 0.0051). No significant correlations between IgG subclasses and Ab-mediated NK cell activation were observed for the placental malaria cohort, suggesting that other Ab features in addition to IgG subclasses may contribute to the modulation of Ab-mediated NK cell activation.

These results show that IgG to DBL2 and DBL3 from malaria-exposed pregnant women can activate NK cells, but that DBL2- or DBL3-specific Ab-mediated NK cell activation does not appear to predict subsequent placental malaria.

### Polyfunctional NK cell activation profiles in pregnant women with malaria

Although levels of individual DBL2- or DBL3-specific NK cell activation markers did not differ the groups of pregnant women with and without placental malaria, it is possible that the proportion of activated NK cells expressing multiple activation markers (“polyfunctional NK cell activation”) differed between the two groups of pregnant women. We therefore assessed the polyfunctional ability of NK cells to secrete TNFα, IFNγ and/or to express CD107a in different combinations. Activated NK cells were selected based on their CD56 and CD16 expression (CD56^dim^CD16^bright^ and CD56^bright^CD16^neg/dim^) (Fig. S5). The levels of CD56 expression have been associated with NK effector function^59^. CD56^bright^ NK cell subsets have been combined here due to low cell numbers, but are mainly characterized by their poor cytotoxic capacity and their high capacity to secrete several types of post-activation cytokines^60^. The CD56^dim^CD16^bright^ NK cell population represents around 90% of peripheral blood NK cells and exhibit potent cytotoxic activity^60,61^.

**Figure 4:**
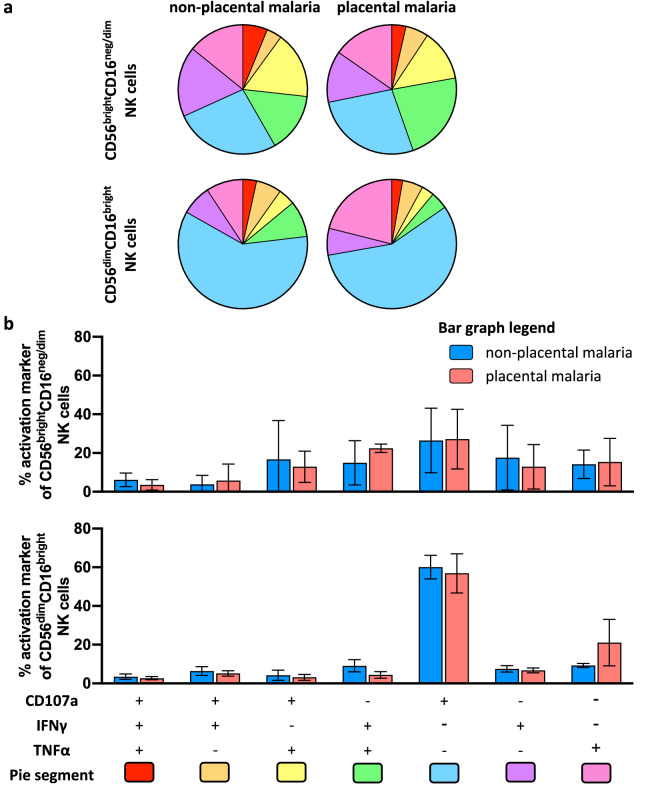
Polyfunctional responses of NK cells induced by DBL2-specific Abs from pregnant women with malaria. NK cells from three separate donors were stimulated with IgG from pregnant women mid pregnancy with non-placental malaria (N = 27), women with placental malaria (N = 50) at delivery in presence of DBL2 and assessed for expression of CD107a, IFNγ and TNFα. (**a**) NK cells were selected based on their CD56 expression (CD56^dim^ and CD56^bright^). Pie and Bar charts show the proportion (**b**) and relative frequency (**c**) of each activation marker combination of only activated NK cells. (**b**) The pie segments correspond to NK cells expressing different combinations of activation markers and are color coded (pie segment legend: pink-red) to indicate increasing polyfunctional NK cell activation. (**c**) The bar graph shows relative frequencies of combinations of activation markers by NK cells stimulated with IgG from non-placental malaria-infected women (blue) or women with placental malaria (red) in presence of DBL2. Mean of three NK cell donors with standard deviation is shown. Statistical analysis between groups was performed using multiple t tests corrected for multiple comparisons using the Holm-Šídák method.

**Figure 5:**
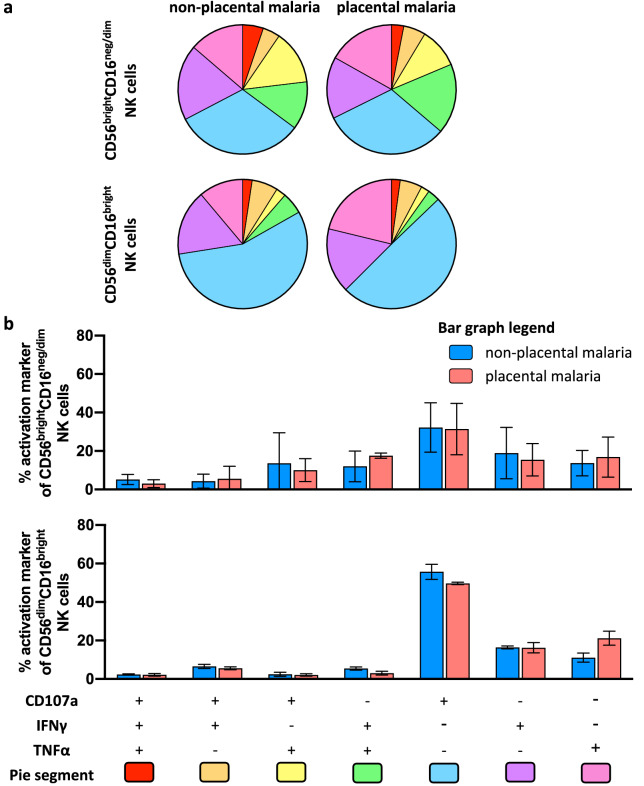
Polyfunctional responses of NK cells induced by DBL3-specific Abs from pregnant women with malaria. NK cells from three separate donors were stimulated with IgG from pregnant women mid pregnancy with non-placental malaria (N = 27), women with placental malaria (N = 50) at delivery in presence of DBL2 and assessed for expression of CD107a, IFNγ and TNFα. NK cells were selected based on their CD56 expression (CD56^dim^ and CD56^bright^). Pie and Bar charts show the proportion (**a**) and relative frequency (**b**) of each activation marker combination of only activated NK cells. (**a**) The pie segments correspond to NK cells expressing different combinations of activation markers and are color coded (pie segment legend: pink-red) to indicate increasing polyfunctional NK cell activation. (**b**) The bar graph shows relative frequencies of combinations of activation markers by NK cells stimulated with IgG from non-placental malaria-infected women (blue) or women with placental malaria (red) in presence of DBL3 (bottom bar graph). Mean of three NK cell donors with standard deviation is shown. Statistical analysis between groups was performed using multiple t tests corrected for multiple comparisons using the Holm-Šídák method.

We observed only a small proportion of CD56^dim^ NK cells which were polyfunctional as indicated by expression of two or more activation markers (DBL2: non-placental malaria: 23.1% of CD56^dim^CD16^bright^ NK cells, placental malaria: 15.4%; DBL3: non-placental malaria: 16.8%, placental malaria: 12.8%). The majority of CD56^dim^CD16^bright^ NK cells were associated with a single function, with a great portion of NK cells exclusively expressing CD107a (Figs. 4 and 5). The smaller subset of CD56^bright^CD16^neg/dim^ NK cells (~ 1–5%) was more polyfunctional, more so for DBL2-specific responses than DBL3 (DBL2: non-placental malaria: 47.7% of CD56^bright^ NK cells, placental malaria: 44.6%; DBL3: non-placental malaria: 35.1%, placental malaria: 36.2%) (Figs. 4 and 5).

The polyfunctional ability of IgG Abs from pregnant women with non-placental malaria or placental malaria to activate NK cells was not significantly different (Figs. 4b and 5b). These findings indicate that IgG Abs against DBL2 and DBL3 from malaria-exposed pregnant women can induce NK cells, with the main response being an upregulation of CD107a expression, but also with a small subset inducing polyfunctional NK cell responses.

### Glycan profiles of IgG Abs in pregnant women with malaria are potentially masked by pregnancy

**Figure 6:**
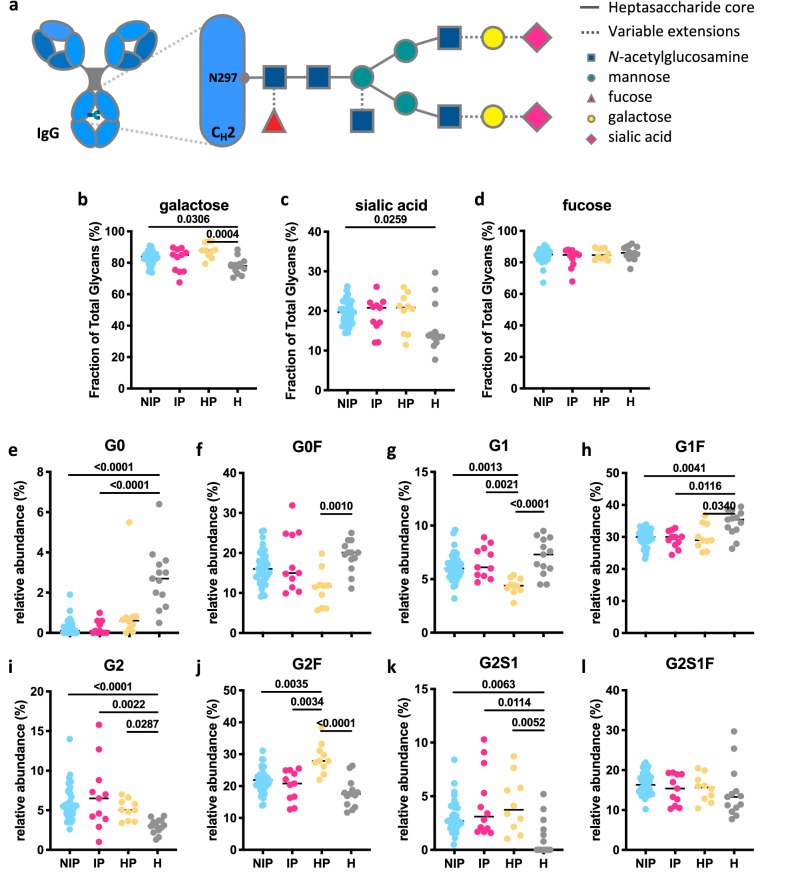
Glycan profiles of IgG Abs in pregnant women with malaria. (**a**) Schematic representation of *N*-linked glycan composition of human IgG Abs. The glycans are attached to asparagine (N) at position 297 in the C_H_2 domain of IgG and have a biantennary heptasaccharide core (solid line) and variable extensions (dash line), such as fucose, galactose and/or sialic acid. Relative abundance of specific types of *N*-linked glycan structures of purified IgG Abs from non-infected pregnant women (NIP; N = 41; blue), pregnant women with malaria infection (IP; N = 11; pink), malaria-naïve healthy pregnant women (HP; N = 10; yellow) and uninfected healthy non-pregnant women (H; N = 13; grey) were profiled. % of Fc glycans with the presence of (**b**) galactose (monogalactosylated or digalactosylated), (**c**) sialic acid and (**d**) fucose. (**e**–**l**) The relative prevalence of several major glycan structures (*G0* agalactosylated, *G1* monogalactosylated, *G2* digalactosylated, *F* fucosylated, *S1* sialylated). Statistical comparison between groups was performed using a Kruskal–Wallis test corrected for multiple comparisons using Dunn’s multiple comparison method (p-values are shown on graphs).

Another mechanism by which the immune system regulates Ab-mediated activation of innate immune cells is the modulation of glycosylation patterns of IgG Abs at a single asparagine residue at position 297 in the C_H_2 domain^36^. *N*-linked glycans are composed of a core complex biantennary structure of mannose and *N*-acetylglucosamine (GlcNAc) with variable additions of sugars such as fucose, galactose, sialic acid and bisecting GlcNAc (Fig. 6a)^36^. These post-translational modifications tune the affinity of IgG Abs for FcγRs, such as FcγRIIIa on NK cells, and regulate effector function^25,62,63^. We evaluated *N*-linked glycosylation patterns of IgG Abs in pregnant women using plasma collected at 14–26 weeks’ gestation. Samples from pregnant women with *P. falciparum* infection (N = 11) and uninfected pregnant women (N = 41) at enrolment were analyzed, together with malaria-naïve healthy pregnant women (N = 10) and non-pregnant women controls (N = 13). *N*-linked glycosylation profiles were analyzed via microchip capillary electrophoresis-laser-induced fluorescence. No statistically significant differences were observed between the two groups of pregnant women (Fig. 6b–l), suggesting that malaria infection in second trimester of pregnancy does not change the total IgG *N*-linked glycan profile. However, comparing *N*-linked glycosylation profiles of IgG from pregnant women, regardless of infection or malaria exposure status, with the profiles of uninfected non-pregnant women, the total IgG of pregnant women exhibited a higher degree of total galactosylation (Fig. 6b, median [IQR]: pregnant non-infected: 83.9% [80.9–86.3%], pregnant infected: 85.0% [75.0–87.4%], pregnant malaria-naïve healthy: 87.90% [86.1–90.6], non-pregnant malaria-naïve healthy: 78.0% [75.2–79.3]) and total sialylation (Fig. 6c, median [IQR]: pregnant non-infected: 19.7% [17.5–22.0%], pregnant infected: 20.8% [16.7–21.8%], pregnant malaria-naïve healthy: 20.8% [17.1–22.3], non-pregnant malaria-naïve healthy: 13.8% [13.3–14.7%]). No differences in total fucosylation were observed. Examining the distribution of specific glycan structures, significantly decreased proportions of G0 and G1F glycan structures were observed in pregnant women compared to non-pregnant women (Fig. 6e,h), whereas elevated proportions of G2 and G2S1, structures were observed in pregnant women compared to non-infected non-pregnant women (Fig. 6i–l). However, differences between malaria-naïve healthy pregnant women and malaria-exposed pregnant women were observed for G1 and G2F glycan structures (Fig. 6g,j), suggesting that malaria-naïve healthy pregnant women have slightly higher anti-inflammatory glycan structures (more galactose and fucose) in comparison to malaria-exposed pregnant women. Placental malaria infections may induce slightly more inflammatory glycan structures within pregnant women. However, no significant differences were observed for total galactose or fucose glycan structures were compared healthy pregnant control (Fig. 6b,d).

*N*-linked glycosylation profiles of purified total IgG from pregnant women at 14–26 weeks of gestation were also assessed to determine if they could predict future clinical outcome (placental malaria status), with no significant differences observed (Fig. S6a–k). We did note that similar differences in *N*-linked glycosylation profiles were maintained between infected pregnant and non-infected non-pregnant women. Furthermore, DBL2- and DBL3-specific Ab-mediated expression of activation markers by NK cells and IgG *N*-linked glycosylation patterns of malaria-exposed pregnant women did not correlate (Fig. S6), except for DBL2-mediated IFNγ production and total fucose (ρ = − 0.4255, p-value = 0.0108) along with a trend for DBL2-mediated CD107a expression and total fucose (ρ = − 0.3744, p-value = 0.0543), and DBL2-mediated TNFα production and total galactose (ρ = 0.3625, p-value = 0.0323). This suggests that the *N*-linked glycosylation profiles of total IgG in pregnant women may be more influenced by pregnancy than by malaria infection.

## Discussion

Naturally acquired immunity to malaria is complex, and likely requires a combination of cell-mediated and humoral immune responses, including the secretion of cytokines, cellular cytotoxicity, and production of functional Abs in order to efficiently clear parasites^18,64^. It has previously been shown that antigen-specific Ab-mediated phagocytosis and engagement with FcγRIIIa on NK cells are linked to protection by the sporozoite-based malaria vaccine RTS,S/AS01^30^. In addition, Ab-dependent NK cell cytotoxicity towards IEs in malaria-exposed individuals can inhibit *P. falciparum* growth^31^. Furthermore, adaptive NK cells, a sub-population of differentiated specialized NK cells, were associated with lower parasitemia and protection against malaria infection through enhanced ADCC response to IEs in the presence of naturally acquired Abs from malaria-resistant individuals^32,65^. The potential of NK cell-mediated ADCC to protect individuals against placental malaria is still to be determined. A limited number of studies have investigated NK cells in the placenta and in the blood at various timepoints during malaria infections^66–69^, however none have considered the implications of ADCC. Here, we demonstrated that Abs generated by pregnant women with malaria from a malaria endemic area in their second trimester against the VAR2CSA subdomains DBL2 or DBL3 were able to induce NK cell activation, but no significant differences in responses were associated with susceptibility to subsequent placental malaria. We observed that the majority of Ab-mediated NK cell activation in women with placental malaria was driven by IgG1, even though a recent study identified malaria-specific IgG1 and IgG3, and engagement with FcγRIIIa (linked to Ab-mediated NK cell activity), as key prediction parameters for protection in malaria RTS,S/AS01 vaccinees^30^. However, even though IgG3 shows high affinity to FcγRs and especially to FcγRIIIa, within this study, it did not correlate with NK cell activation in pregnant women. This suggests that IgG subclass distribution may not be the only factor that modulates NK cell activation during pregnancy, and that other IgG features, such as *N*-linked glycans may impact Ab-mediated NK cell activation. Currently, the importance of VAR2CSA-specific Ab-mediated responses in protection from placental malaria is unclear, as Ab responses to recombinant VAR2CSA antigens at delivery are associated with the presence of placental infection, and may represent markers of infection, rather than correlates of protection^14^. In addition, there is no malaria-specific antigen, which can be used as a universal antigenic control to assess blood stage parasite Ab-mediated NK cell responses.

Within our study, Ab-mediated activation of NK cells was largely associated with the upregulation of CD107a expression, a surrogate marker of ADCC activity^56,70^, with smaller fractions of activated NK cells producing only IFNγ and TNFα or in combination with CD107a, suggesting that the majority of Ab-activated NK cells were potentially cytotoxic in the absence of inflammation. We speculate that this balance of Ab-mediated activation may be beneficial, as excessive secretion of pro-inflammatory cytokines, such as IFNγ and TNFα, in the placenta of malaria-infected women, especially in primigravidae, has been associated with placental pathology and adverse clinical outcomes^27,71^. Placental malaria is associated with activation of pro-inflammatory host cells, such as monocytes and macrophages causing inflammation of the placenta^8,72^. Our findings suggest that Ab-mediated activation of NK cells, potentially does not contribute to the overproduction of pro-inflammatory cytokines and resulting pathologies. Several studies suggest that progesterone, estrogen and cortisol dampen NK cell cytotoxic activities during pregnancy^73–75^. However, the increased concentration of cortisol during pregnancy could also inhibit NK cell activity against *P. falciparum* IEs^76^. Here, NK cells purified from whole blood of malaria-naïve healthy donors were used instead of pregnant women with malaria, and effects of pregnancy-associated hormones were not represented by non-pregnant malaria-naïve healthy donor blood NK cells.

We would like to acknowledge limitations of our study. NK cells from healthy malaria-naïve donors were used instead of pregnant women with malaria. This could have skewed for specific NK cell subsets, which may be underrepresented during pregnancy. Our work studied peripheral NK cells which may have substantially different responses to uterine NK cells and adaptive NK cells. Uterine NK cells are functionally different, do not circulate outside the uterus, and are more difficult to access for functional studies^77^. The analysis of peripheral NK cells is however relevant in that they can access the site of infection, the syncytiotrophoblast cell surface lining of the maternal peripheral blood^8^. Nevertheless, in future studies, parasite loads in the placenta at birth, or other clinical markers of disease severity, could also be considered, but this information was not collected for the majority of individuals in this cohort.

One limitation regarding the plate-bound Ab-mediated NK cell assay is that it does not mimic the interaction between NK cell and IEs as shown before^31,78^. However*,* our study is complementary to current placental malaria vaccine studies, which also only use DBL2 antigens^33,34^, and in vitro NK cell ADCC assays could be used for high-throughput screens of serum samples^32^. Furthermore, additional activation markers of NK cell subpopulations, such as CD57, CD25, CD69 or the inhibitory receptor programmed death-1 (PD-1) could be considered for future studies^23,78–80^.

IgG *N*-linked glycosylation profiles can influence the engagement of IgG Abs with FcγRIIIa on NK cells^36^. Surprisingly, we did not observe any differences in Fc *N*-linked glycan profiles between pregnant women infected with *P. falciparum* and their non-infected counterparts. Consistent with previous studies^45,46^, we observed a higher degree of galactosylation and sialylation of IgG Abs from pregnant women, regardless of malaria infection, compared to non-pregnant women. Overall, these results suggest that anti-inflammatory Fc *N*-linked glycans are elevated in both healthy and malaria-exposed pregnant women, which may dampen the Ab-mediated activation of NK cells in pregnant women with malaria infection. These changes have been associated with a less inflammatory profile during pregnancy. Fc *N*-linked glycan patterns of IgG Abs can be globally modulated during the course of inflammation, autoimmune disease or pregnancy^46,81^. For example, in patients with lupus erythematosus or rheumatoid arthritis (RA), reduced galactosylation and sialylation of IgG Abs correlates with pro-inflammatory immune responses and disease severity^82^. Intriguingly, the majority of pregnant women with RA undergo pregnancy-induced remission, which occurs simultaneously with the upregulation of IgG galactosylation and sialylation, such that inflammatory RA-associated glycosylation patterns are masked by pregnancy^83–85^. We observe a similar increase in galactosylation and sialylation of the IgG Abs in pregnant women, regardless of malaria infection status^46,81^. We acknowledge that the malaria-naïve healthy pregnant women in our study are more progressed in their pregnancy, which could affect the glycosylation profiles. However, these Fc *N*-linked glycans may explain why secretion of pro-inflammatory cytokines was suppressed from NK cells within our assays.

In healthy pregnancy, highly galactosylated Abs may be more effectively transferred across the placenta and may be able to mediate CD107a degranulation of both maternal and cord NK cells^86^. The transfer of maternal Abs across the placenta is mediated by binding to the neonatal Fc receptor (FcRn), which is a key process for neonatal immunity, as neonates cannot sufficiently generate IgG Abs^87^. However, contradictory roles for IgG glycosylation on FcRn binding have been reported^86,88,89^, including studies which show significant Ab galactosylation-driven changes in FcRn affinity and NK cell-activating Abs are selectively transferred across the placenta^86,88^, while another study showed that placental IgG transport is not Fc glycosylation selective^89^. In addition, Jennewein et al*.* considered transfer of maternal Abs across the placenta via binding to FcRn and FcγRIIIa, while other more recent studies suggest that FcγRIIIa do not play a role in maternal–fetal Ab exchange^87^. Defining the mechanisms of placental transfer, including the role of Fc glycosylation, may offer novel insights for the rational development of maternal vaccines to enhance transfer of protective Abs to fetuses and reduce their vulnerability^86,90^, and should be considered in the further development of vaccine candidates.

A limitation of capillary electrophoresis-laser-induced fluorescence is that not all Fc *N*-linked glycan profiles are clearly detectable. However, these additional patterns make up only a small fraction of human IgG Fc *N*-linked glycans^91^, and more sensitive techniques such as liquid chromatography mass spectrometry require extensive protein clean up and in-solution digestion, in-depth proteome and glycoform analysis^92^. Furthermore, the evaluation of DBL2- and DBL3-specific IgG glycosylation profiles would allow us to more accurately assess the contribution of *N*-linked glycans to Ab-mediated NK cell activation, unfortunately these assays require large volumes of plasma samples^25,93^, which were not available for this cohort. Previous studies examining antigen-specific *N*-linked glycosylation of IgG from HIV-infected pregnant women have observed significantly different profiles between HIV, tetanus and pertussis toxin specific-IgG^94^, thus future studies where adequate sample is available should assess for malaria-specific IgG glycan patterns. Vaccine studies assessing healthy non-pregnant volunteers have demonstrated that antigen-specific IgG glycosylation profiles can be modulated by vaccination^44^. It is still unclear if antigen-specific IgG glycosylation profiles can be modulated in pregnant women, or if pregnancy-associated global glycan changes will mask any antigen-specific glycosylation effects as observed in RA^83–85^. Determining if antigen-specific Ab glycosylation patterns are associated with clinically relevant outcomes of placental malaria could inform the design of the next generation of maternal vaccines^95^. Overall, our study highlights the necessity to better understand Ab effector functions, such as Ab-mediated NK cell activation, and the potential effect of *N*-linked glycans modulation during pregnancy upon protection from, or susceptibility to, malaria and other infectious diseases.

## Methods

### Study participants

During a randomized controlled trial of Intermittent Preventive Treatment in Pregnancy (IPTp), plasma samples were collected from pregnant women in Madang Province, Papua New Guinea (PNG) between November 2009 and August 2012^96^. Plasma samples were obtained upon enrolment into the prospective study at 14–26 gestation weeks, when pregnant women presented at the hospital for their first medical examination and were stored at − 80 °C. Infection status of pregnant women was determined at collection time point by light microscopy of Giemsa-stained peripheral blood smears (with malaria, N = 11; without malaria, N = 41, 25 pregnant women had unknown malaria infection status at sample collection).

In this cross-sectional study, parasitemia status was determined at delivery by light microscopy of Giemsa-stained peripheral blood smears, as well as by polymerase chain reaction of peripheral blood at delivery^97^. Samples were categorized based on the presence of *P. falciparum* parasites in peripheral blood at delivery. Groups included women who were positive for *P. falciparum* IEs in the placenta (placental malaria, N = 50) or women who were positive for *P. falciparum* IEs in peripheral blood but did not show any sequestering of IEs in the placenta (non-placental malaria, N = 27) (Fig. 1). The groups were frequency matched for primigravidae, age, bed net use, rural residency and type of malaria preventive treatment received (Table 1). Ethical approval was obtained from the PNG Institute of Medical Research Institutional Review Board, the PNG Medical Research Advisory Council, and the Melbourne Health Human Research Ethics Committee.

**Table 1:** Characteristics of study participants.

Characteristic	Placental malaria N = 50	Non-placental malaria N = 27
Age (years)	24.6 [5.24]	23.7 [5.05]
**Gravidity**		
Primigravidae	29 (58)	14 (51.9)
Secundigravidae	7 (14)	8 (29.6)
Multigravidae	14 (28)	5 (18.5)
Mean gestational age (days)	147.5 [31.3]	152.2 [19.8]
Mean maternal weight (kg)	53.5 [8.2]	54.1 [7.4]
Mean maternal height (cm)	154.6 [6.9]	148.4 [32]
**IPTp regime**		
SPAZ	20 (40.0)	9 (33.3)
SPCQ	30 (60.0)	18 (66.7)
**Residence**		
Urban	6 (12)	2 (7.4)
Periurban	4 (8)	7 (25.9)
Rural	38 (76)	18 (66.7)
Migrant	2 (4)	0 (0)
Bed net use	40 (80)	21 (77.8)

Data shown as mean [standard deviation], or number (%).

*IPTp* intermittent preventive treatment in pregnancy, *SPAZ* sulphadoxine-pyrimethamine and azithromycin, *SPCQ* sulphadoxine-pyrimethamine + chloroquine.

Plasma samples from malaria-naïve healthy Melbourne donors (N = 8) were chosen as negative controls, because many matched women from PNG would have been exposed to malaria and skewed negative responses. For the Fc *N*-linked IgG glycan profiling, samples of malaria-naïve healthy non-pregnant women (N = 13) were used (age: 34.3 ± 7.7 years). Plasma samples from individual healthy Melbourne donors were obtained in accordance with the University of Melbourne Human ethics approval (#1443420) and the Australian National Health and Medical Research Council Statement on Ethical Conduct in Human Research. Samples from malaria-naïve healthy pregnant women at the end of their second/beginning of their third trimester (N = 10) were obtained to compare pregnancy-specific Fc *N*-linked IgG glycan profiles (age: 31.3 ± 2.8 years; mean gestational age: 196 ± 4 days). Ethical approval was granted by the Mercy Health Board Human Research Ethics Committee (R10/16). All participants provided written informed consent.

### IgG antibody purification

IgG Abs were purified from plasma of pregnant women at enrolment according to manufacturer’s protocol via Melon Gel chromatography (Melon Gel IgG Purification Kit, Thermo Fisher Scientific, USA)^98^. IgG Ab samples were centrifugated through 100 kDa Amicon Ultra filters (Merck & Co, USA) at 14,000 × *g* for 10 min to remove excess albumin proteins and buffer exchanged into phosphate buffered saline (PBS). The IgG concentration and purity were quantitated using a human IgG ELISA development kit (Mabtech AB, Sweden). The IgG Ab samples were diluted in PBS to adjust Ab concentration to 0.25 mg/ml for Ab-dependent NK cell activation assays and 2 mg/ml for *N*-linked glycan profiling. The samples were stored at − 20 °C until further use.

### Natural killer cell isolation

NK cells were isolated from heparinized whole blood from malaria-naïve healthy donors with RosetteSep (Stemcell Technologies, Canada) and density gradient separation via Ficoll (Bio-Strategy Lab, Australia) centrifugation according to manufacturer’s protocols.

### Antibody-dependent natural killer cell activation

Ab-dependent plate-bound NK cell activation assays were modified for use with DBL antigens^24–26,99^. In order to compare NK cell activation induced by DBL domains and controls, various IgG Ab (0.0625–0.25 mg/ml) and protein (50–300 ng/well) concentrations were tested. Purified IgG was used to control for IgG concentration for each individual sample and to ensure no other components in plasma contributed to activation of NK cells. Concentrations of 200 ng of protein/well and 0.25 mg/ml of IgG Abs were chosen to test individual samples.

High protein-binding plates (NUNC MaxiSorp flat bottom; Thermo Fisher Scientific) were coated with either DBL2^33^ or DBL3^100^ (200 ng/well) at 4 °C for 12 h. Bovine serum albumin (BSA; Sigma-Aldrich, USA) was used to control for unspecific binding. Simian immunodeficiency virus (SIV) envelope protein gp120 (Sino Biological Inc., China) and influenza hemagglutinin (H)3 (A/Switzerland/9715293/2013; Immune Technology Corp., USA) were used as negative and positive antigen controls, respectively. H3 was selected as a universal technical control, as all individuals have been previously exposed to influenza, and Abs to H3 are highly cross reactive and strong inducers of NK cell activation^24^.

After washing with PBS, the plate was blocked with 1% PBS-BSA for 1 h. Purified IgG (0.25 mg/ml) was added to each well and incubated at 37 °C for 2 h. NK cells (0.25 × 10^6^ cells/ml) were then incubated with anti-CD107a-APC-H7 (BD, USA), brefeldin A (10 mg/ml; Sigma-Aldrich) and GolgiStop (BD) for 5 h at 37 °C. NK cells were stained for surface markers using anti-FcγRIII–BV605 (BD), anti-CD56-BUV737 (BD), anti-FcγRII-APC (BioLegend, USA) and anti-CD3-PerCP (BD), and intracellularly with anti-IFNγ-PE (BD) and anti-TNFα-BV785 (BD) via fixation with 10% paraformaldehyde and Perm B solutions (Thermo Fisher Scientific). NK cells were analyzed via flow cytometry and combination gates in FlowJo (BD) were used to include all NK cells expressing activation marker CD107a (degranulation marker), IFNγ and TNFα (cytokines). Mean fluorescence intensity (MFI) of NK cells incubated without Abs was subtracted as background to determine Ab-mediated activation and median of SIV gp120-specific responses was subtracted as non-specific NK cell activation.

### Multiplex assays of antibodies binding to Duffy binding-like domains

DBL2 and DBL3 domains were coupled to Bio-Plex magnetic carboxylated microspheres (Bio-Rad, Hercules, USA) as per manufacturer’s instructions. The antigen-coupled microspheres were resuspended in storage buffer (PBS, 0.05% sodium azide), and stored in the dark at 4 °C for immediate use. Their concentration was determined using a hemocytometer.

The DBL-coupled microspheres were mixed, resuspended in 1% PBS-BSA and added to wells of a 96-well round bottom plate (Greiner Bio-One, Kremsmünster, Austria) containing plasma in a 1:100 dilution in PBS. The sealed plates were incubated on a plate shaker overnight at 4 °C. After incubation, the plates were centrifuged and washed with PBS-0.1% Tween using a magnetic plate-washer (Bio-Plex Pro wash station, Bio-Rad). The anti-human Ab (total IgG, IgG1, IgG2, IgG3, IgG4) detectors conjugated with phycoerythrin (PE; all SouthernBiotech, Birmingham, USA) were added and the mixture was incubated for 2 h on a plate shaker. After washing with PBS and resuspending in xMAP drive fluid (Life Technologies, Carlsbad, USA), the plates were read on a Bio-Plex MAGPIX multiplex reader (Bio-Rad), and analysed using Bio-Plex Manager software (Bio-Rad). The median fluorescence intensity is directly proportional to the amount of Ab bound to the antigens^101^.

### IgG *N*-linked glycan profiling

*N*-linked glycan profiles of purified IgG Abs (2 mg/ml) were measured on the LabChip GXII Touch instrument (PerkinElmer, USA) according to the ProfilerPro glycan profiling LabChip GXII Touch protocol. Microchip capillary electrophoresis-laser-induced fluorescence analysis of digested and labelled *N*-linked glycans was performed. The relative prevalences of several glycan profiles of IgG Abs were analyzed using the LabChip GX Reviewer (PerkinElmer) software. Peaks were assigned based on migration of known standards and glycan digests^91^. Peak area and therefore the relative prevalence of each glycan pattern was calculated.

### Statistical methods

Statistical analyses were performed in Prism version 8 (GraphPad, USA). Statistical comparison of NK cell activation markers between groups was performed using Kruskal–Wallis test with Dunn’s multiple comparison method. Statistical comparison between groups for the analysis of activated NK cells polyfunctionality was performed using multiple t tests corrected for multiple comparisons using the Holm-Šídák method. Spearman's rank correlation coefficients of antigen binding and NK cell activation were calculated. Kruskal–Wallis tests with Dunn’s multiple comparison method were conducted to determine the significance of differences observed in glycan prevalence between pregnant women and their non-pregnant counterparts. Statistical significance was considered when p-values were less than 0.05.

## Supplementary Information

Supplementary Figures.

## Data Availability

Derived data supporting the findings of this study are available from the corresponding author upon request.
