# Supplementary material for: Antibody mediated activation of natural killer cells in malaria exposed pregnant women
Source: Sci Rep. 2021 Feb 18;11:4130. doi: 10.1038/s41598-021-83093-4 (PMC7893158; doi:10.1038/s41598-021-83093-4)
Supplement: Supplementary file 1 — Supplementary Figures. [file 41598_2021_83093_MOESM1_ESM.pdf]

Antibody mediated activation of Natural Killer cells in malaria exposed pregnant women

Timon Damelang[1], Elizabeth H. Aitken[2], Wina Hasang[2], Ester Lopez[1], Martin Killian[1,3,4], Holger W. Unger[5,6,7], Ali Salanti[8,9], Alexis Shub[10], Elizabeth McCarthy[10], Katherine Kedzierska[1], Martha Lappas[10], Stephen J. Kent[1,11], Stephen J. Rogerson[2], and Amy W. Chung[1*]

[1]Peter Doherty Institute for Infection and Immunity, Department of Microbiology and Immunology, University of Melbourne, Melbourne, Victoria, Australia
[2]Peter Doherty Institute for Infection and Immunity, Department of Medicine, University of Melbourne, Melbourne, Victoria, Australia
[3]Department of Internal Medicine, Centre Hospitalier Universitaire de Saint-Etienne, Saint-Etienne, France
[4]Groupe sur l'Immunité des Muqueuses et Agents Pathogènes, Université Jean Monnet Saint-Etienne, Saint-Etienne, France
[5]Liverpool School of Tropical Medicine, Liverpool, United Kingdom
[6]Department of Obstetrics and Gynaecology, Royal Darwin Hospital, Darwin, Northern Territory, Australia
[7]Menzies School of Health Research, Charles Darwin University, Darwin, Northern Territory, Australia
[8]Centre for Medical Parasitology, Department for Immunology and Microbiology, University of Copenhagen, Denmark
[9]Copenhagen University Hospital, Department of Infectious Disease, Copenhagen, Denmark.
[10]Department of Obstetrics & Gynaecology, University of Melbourne, Melbourne, Victoria, Australia
[11]Melbourne Sexual Health Centre, Infectious Diseases Department, Alfred Health, Monash University, Melbourne, Victoria, Australia

*** Correspondence:**
Amy W. Chung
awchung@unimelb.edu.au

Figure S1

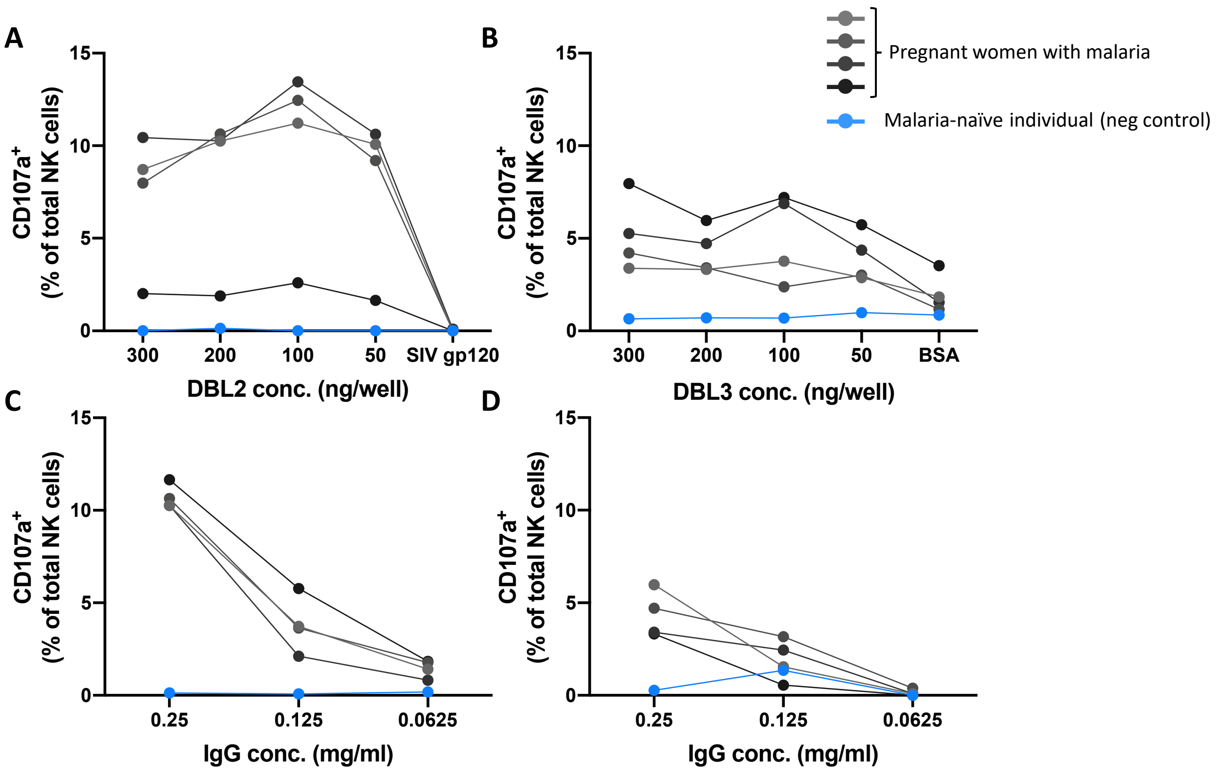

Figure S1. Optimization of Ab-dependent plate-bound NK cell activation assays for use of DBL domains. Optimal antigen **(A-B)** and IgG concentrations **(C-D)** were tested for Ab-dependent plate-bound NK cell activation assays. For comparison **(A)** & **(C)** are normalized to same condition (200ng DBL2 and 0.25mg/ml IgG) and **(B)** & **(D)** are normalized to same condition (200ng DBL3 and 0.25mg/ml IgG). IgG from four pregnant women with malaria (shades of grey) and a malaria-naïve individual (blue) are shown.

Figure S2

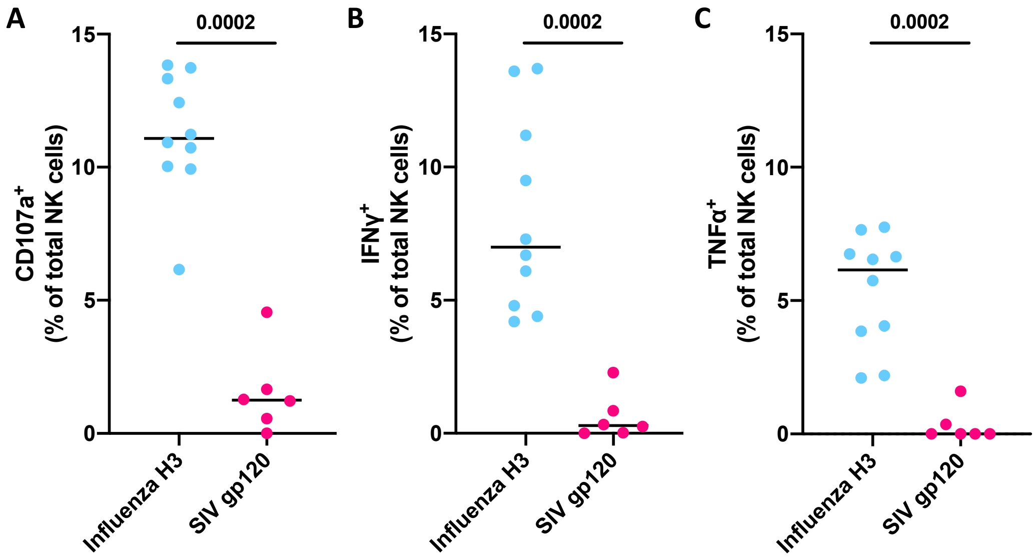

Figure S2. Validation of Ab-dependent plate-bound NK cell activation assays using control antigens. NK cell activation assays were validated using IgG Abs from malaria-naïve individuals in the presence of influenza hemagglutinin (H3) (positive control; light blue) and SIV gp120 (negative control; pink) proteins (200ng and 0.25mg/ml IgG). NK cells were assessed for surface **(A)** CD107a expression and intracellular **(B)** IFNγ and **(C)** TNFα production. Significance between groups was analyzed using Mann-Whitney U tests (p-values are shown on graphs).

Figure S3

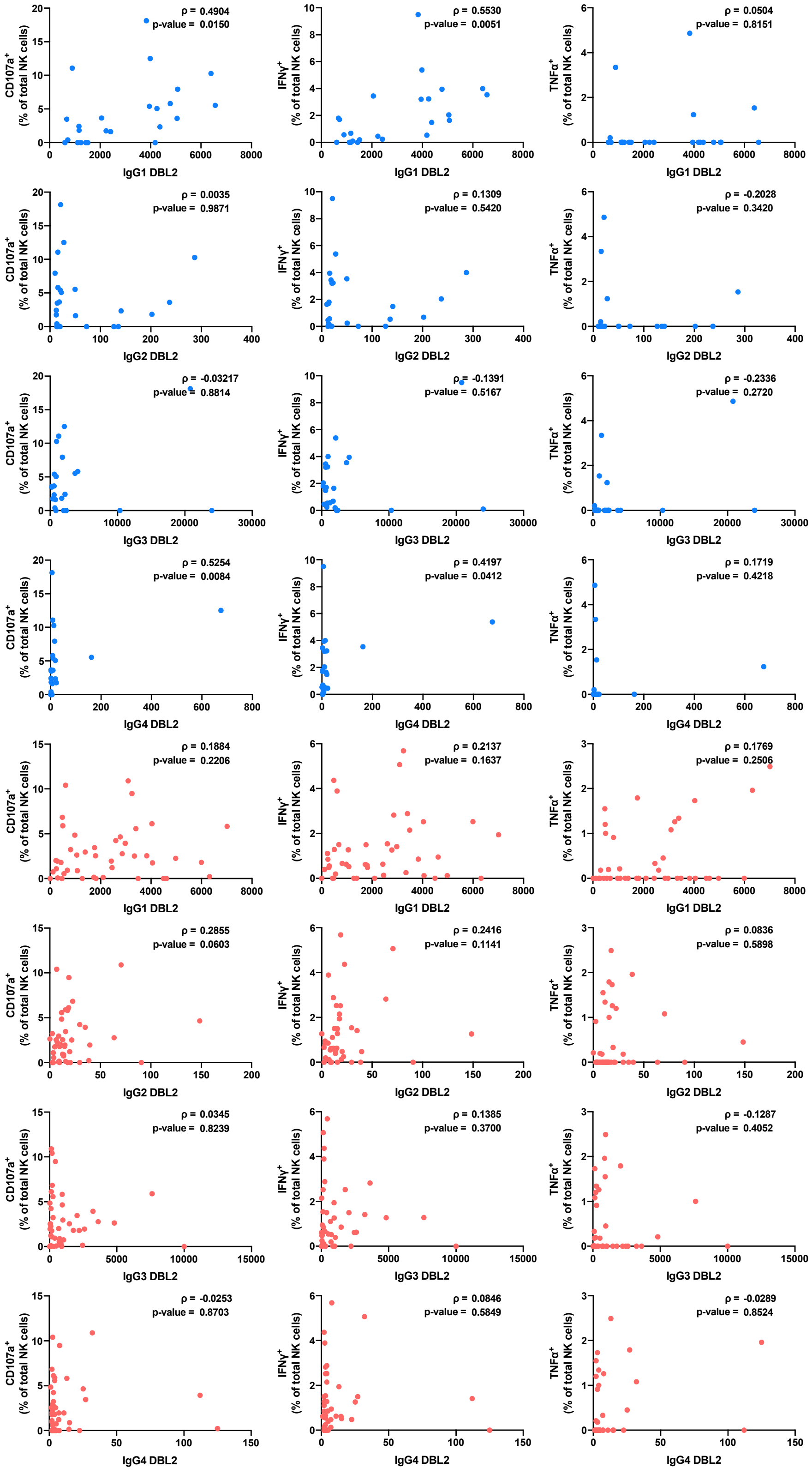

Figure S3. Correlations between DBL2-binding capacity of IgG Abs and expression of activation markers by NK cells. DBL2-binding capacity of IgG1, IgG2, IgG3 and IgG4 Abs from pregnant women with non-placental malaria (N=27; blue) and placental (N=50; red) was measured in multiplex assays. Spearman's rank correlation coefficients (ρ values and p-values) between antigen binding and expression of CD107a, IFNγ and TNFα by Ab-activated NK cells are shown.

Figure S4

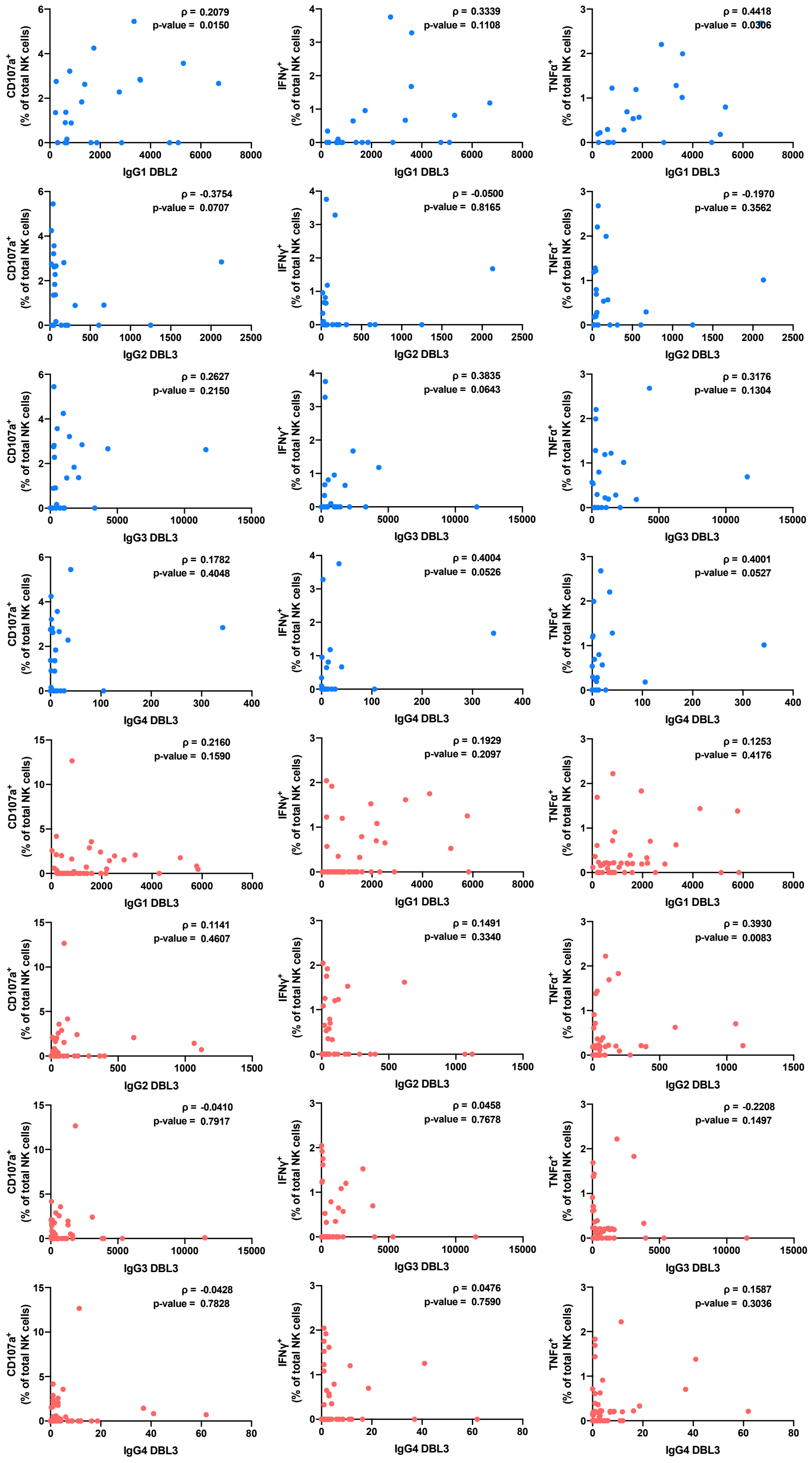

Figure S4. Correlations between DBL3-binding capacity of IgG Abs and expression of activation markers by NK cells. DBL3-binding capacity of IgG1, IgG2, IgG3 and IgG4 Abs from pregnant women with non-placental malaria (N=27; blue) and placental (N=50; red) was measured in multiplex assays. Spearman's rank correlation coefficients (ρ values and p-values) between antigen binding and expression of CD107a, IFNγ and TNFα by Ab-activated NK cells are shown.

Figure S5

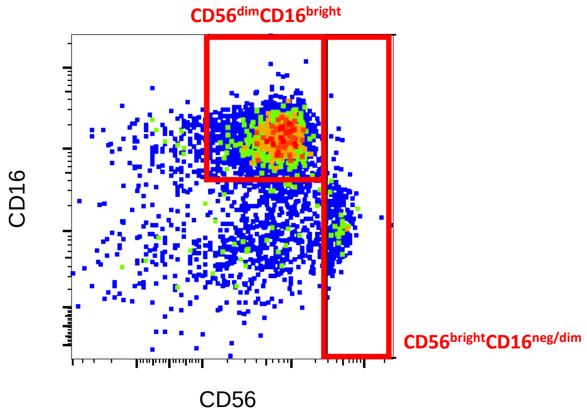

Figure S5. Subpopulations of NK cells selected for polyfunctional analysis based on the relative expression of CD56 and CD16. CD56^{dim}CD16^{bright} were gated; CD56^{bright}CD16^{neg} and CD56^{bright}CD16^{dim} were combined due to low cell numbers.

Figure S6

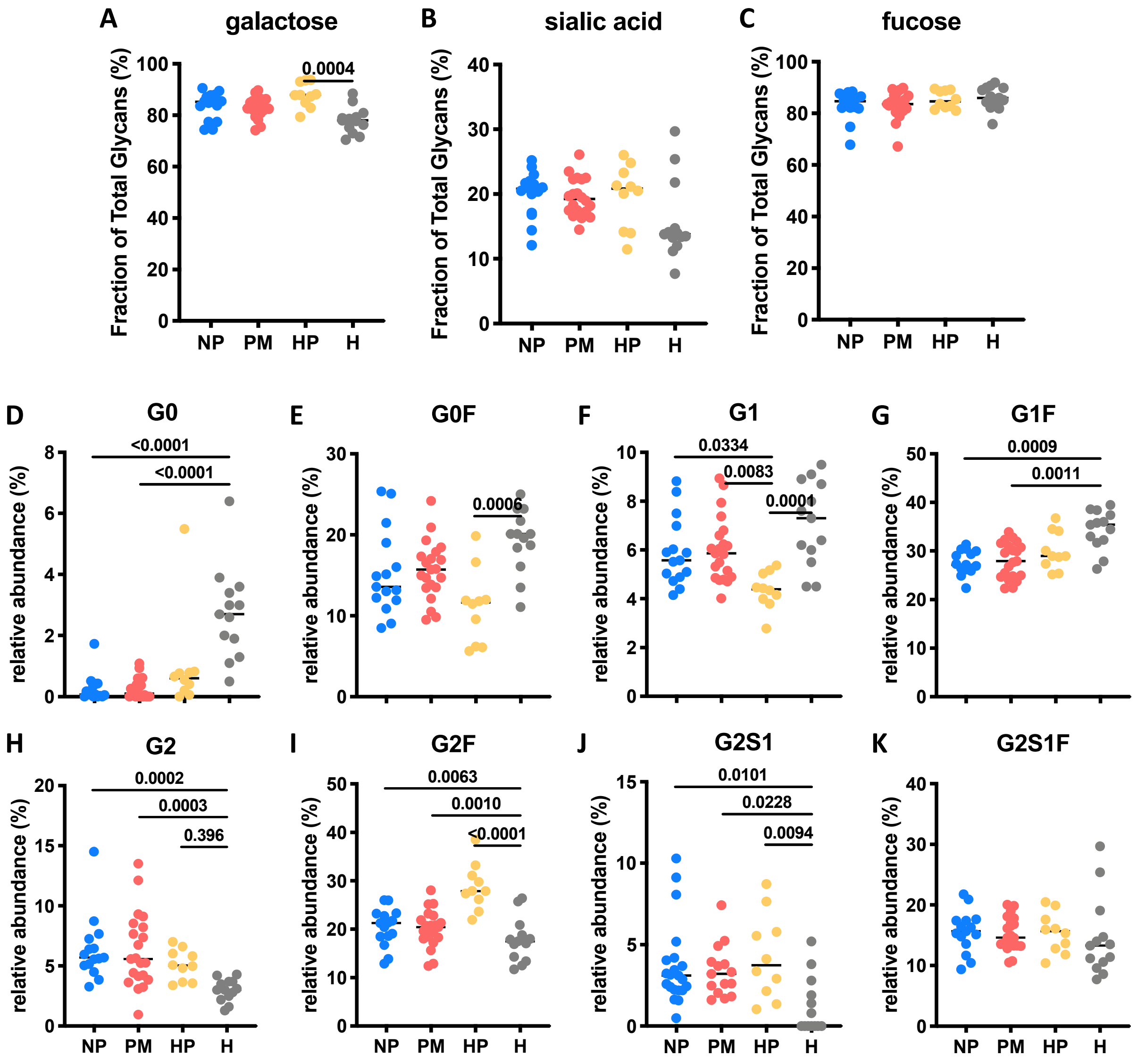

Figure S6. Glycan profiles of IgG Abs in pregnant women with malaria. Specific types of *N*-linked glycan structures of purified IgG Abs from pregnant women were profiled based on clinical outcome: non-placental malaria (NP; N=21; blue) and placental malaria (PM; N=15; red) in comparison to malaria-naïve healthy pregnant women (HP; N=10; yellow) and uninfected healthy non-pregnant women (H; N=13; grey). **(A-C)** Fraction of **(A)** galactose, **(B)** sialic acid and **(C)** fucose of total glycans are shown. **(D-K)** The relative prevalences of several major glycan patterns are graphed (G0, agalactosylated; G1, monogalactosylated; G2, digalactosylated; F, fucosylated; S1, sialylated). Statistical comparison between groups was performed using a Kruskal-Wallis test corrected for multiple comparisons using Dunn's multiple comparison method (p-values are shown on graphs).

Figure S6

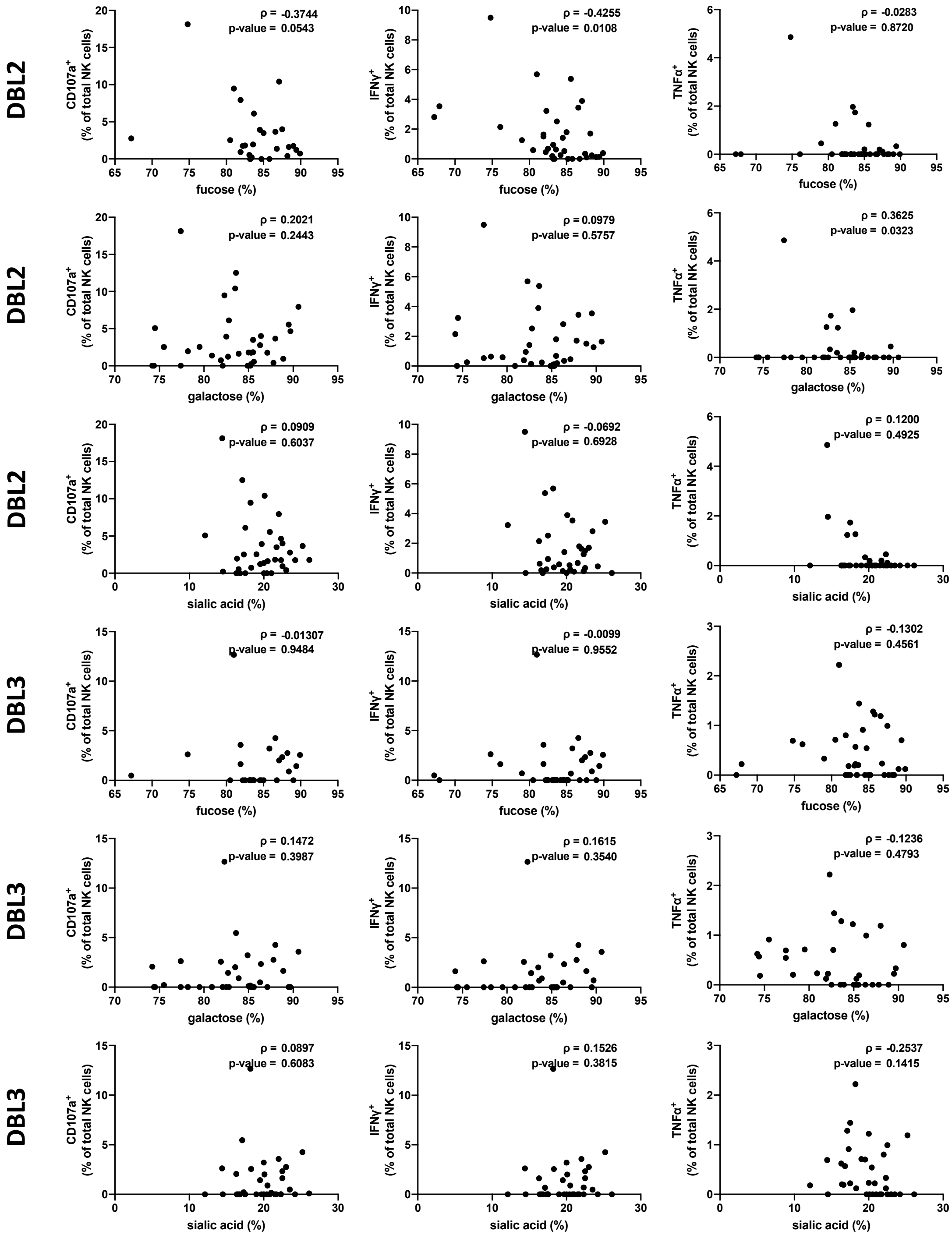

Figure S6. Correlations between DBL2- and DBL3-specific Ab-mediated NK cell activation and IgG *N*-linked glycosylation patterns of malaria-exposed pregnant women. Spearman's rank correlation coefficients (ρ values and p-values) of DBL2- and DBL3-specific Ab-mediated expression of activation markers (CD107a, IFNγ and TNFα) and IgG *N*-linked glycosylation patterns (fucose, galactose and sialic acid) from pregnant women exposed to malaria are shown.
